# Recurrent Pneumothorax Complicated by Pleuroparenchymal Fibroelastosis Successfully Treated With Pleurodesis Using 50% Glucose: A Case Report

**DOI:** 10.7759/cureus.109896

**Published:** 2026-05-29

**Authors:** Yusuke Nabe, Hiroshi Mizuuchi, Masaaki Inoue, Junichi Yoshida

**Affiliations:** 1 Department of Chest Surgery, Shimonoseki City Hospital, Yamaguchi, JPN

**Keywords:** 50% glucose, pleurodesis, pleuroparenchymal fibroelastosis, pneumothorax, postoperative recurrence

## Abstract

Pleuroparenchymal fibroelastosis (PPFE), a rare interstitial lung disease primarily affecting the upper lung lobes, leads to pleural thickening and subpleural fibroelastosis. Patients with PPFE are often prone to recurrent pneumothorax because their lung tissues are fragile and do not expand properly. Here, we report a case of recurrent pneumothorax associated with PPFE that was successfully treated with pleurodesis using a 50% glucose solution. A 66-year-old non-smoking woman presented with a right pneumothorax and underwent thoracoscopic bullectomy after imaging revealed a pulmonary bulla and bilateral apical infiltrates. PPFE was confirmed via histopathological examination. Although the initial recovery after surgery was uneventful, the pneumothorax recurred on postoperative day 16. The patient declined repeat surgery, and pleurodesis was performed using a 50% glucose solution because of her minocycline allergy. Following the procedure, she developed transient pleural effusion, which subsequently resolved; lung re-expansion was achieved, and no recurrence was observed on follow-up imaging. This case highlights that glucose pleurodesis may be a safe and effective alternative treatment for recurrent pneumothorax in patients with PPFE when conventional therapies are unsuitable.

## Introduction

Pleuroparenchymal fibroelastosis (PPFE) is a rare interstitial lung disease characterized by lesions in the upper lobes, with excessive elastic fibrosis of the pleura and adjacent subpleural parenchyma [1]. In a 2013 American Thoracic Society/European Respiratory Society official statement, the disease was described as a group of rare idiopathic interstitial pneumonias [2]. PPFE accounts for less than 1% of all interstitial lung diseases and may be associated with bilateral upper lobe pneumothorax [3]. It is caused by hardened, fragile subpleural tissue and was reported to be associated with recurrent or persistent pneumothorax in 75% (33/44) of cases in a previous study [4]. Chronic reduction in the upper lobe volume and flattening of the thoracic wall impair ventilation and perfusion and worsen subpleural fibrosis [5]. PPFE is considered idiopathic [1] or associated with multiple factors (e.g., other fibrous interstitial lung diseases, hematopoietic stem cell transplantation, solid organ transplantation, recurrent lung infections, autoimmune diseases, past chemotherapy, or occupational exposure) [5,6].

Symptoms of PPFE include progressive dyspnea, coughing, and chest tightness [3]. The main diagnostic criteria are upper lobe pleural thickening and associated subpleural fibrosis on chest computed tomography (CT) and pathological findings of pleural fibrosis, followed by intra-alveolar fibrosis and alveolar septal elasticity [7]. The only curative treatment for PPFE is lung transplantation, which is not feasible for most older patients or those with comorbidities [6]. There is no disease-specific treatment, and treatment mainly consists of palliative therapy focused on symptom control [3]. Lung function gradually declines in most patients with PPFE, leading to respiratory failure and ultimately, death [5,6,8]. The median overall survival after diagnosis is 35.3 months [4], indicating a poor prognosis. Pleurodesis using a 50% glucose solution is performed for postoperative lung fistulas and spontaneous pneumothorax [9,10]. The mechanism by which pleurodesis occurs may be related to osmotic pressure differences [11], but it is not yet fully understood.

Herein, we report a rare case of recurrent pneumothorax complicated by PPFE after thoracoscopic right lung bullectomy that was cured by pleurodesis with 50% glucose.

## Case presentation

**Figure 1:**
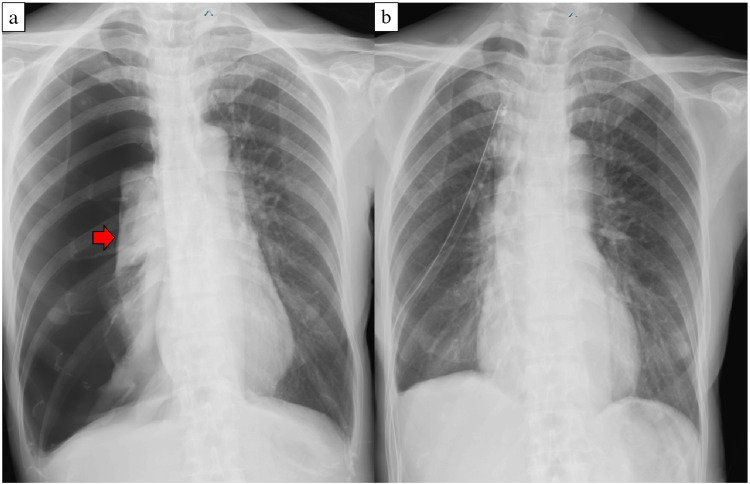
Chest radiographs at the time of initial examination a) The right lung is severely collapsed (red arrow). The mediastinum is displaced to the left, and a diaphragmatic depression is observed; b) After insertion of the right chest tube. The expansion of the right lung improved.

**Figure 2:**
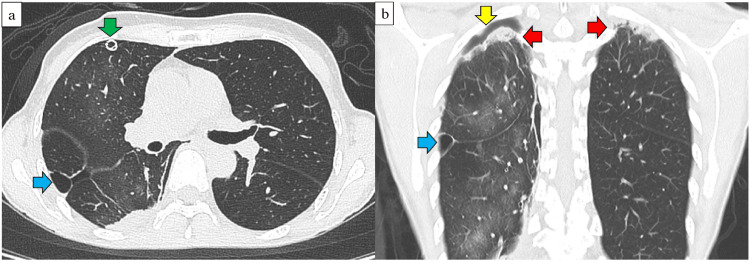
Computed tomography at the initial examination a) After insertion of right chest tube (horizontal section). A cyst (blue arrow) is observed between the right middle and lower lobes; b) After insertion of the right chest tube (coronal section). Infiltrative shadows (red arrows) are observed in both lung apices. An air space (yellow arrow) is observed in the right lung apex. A cyst (blue arrow) is observed between the right middle and lower lobes.

**Figure 3:**
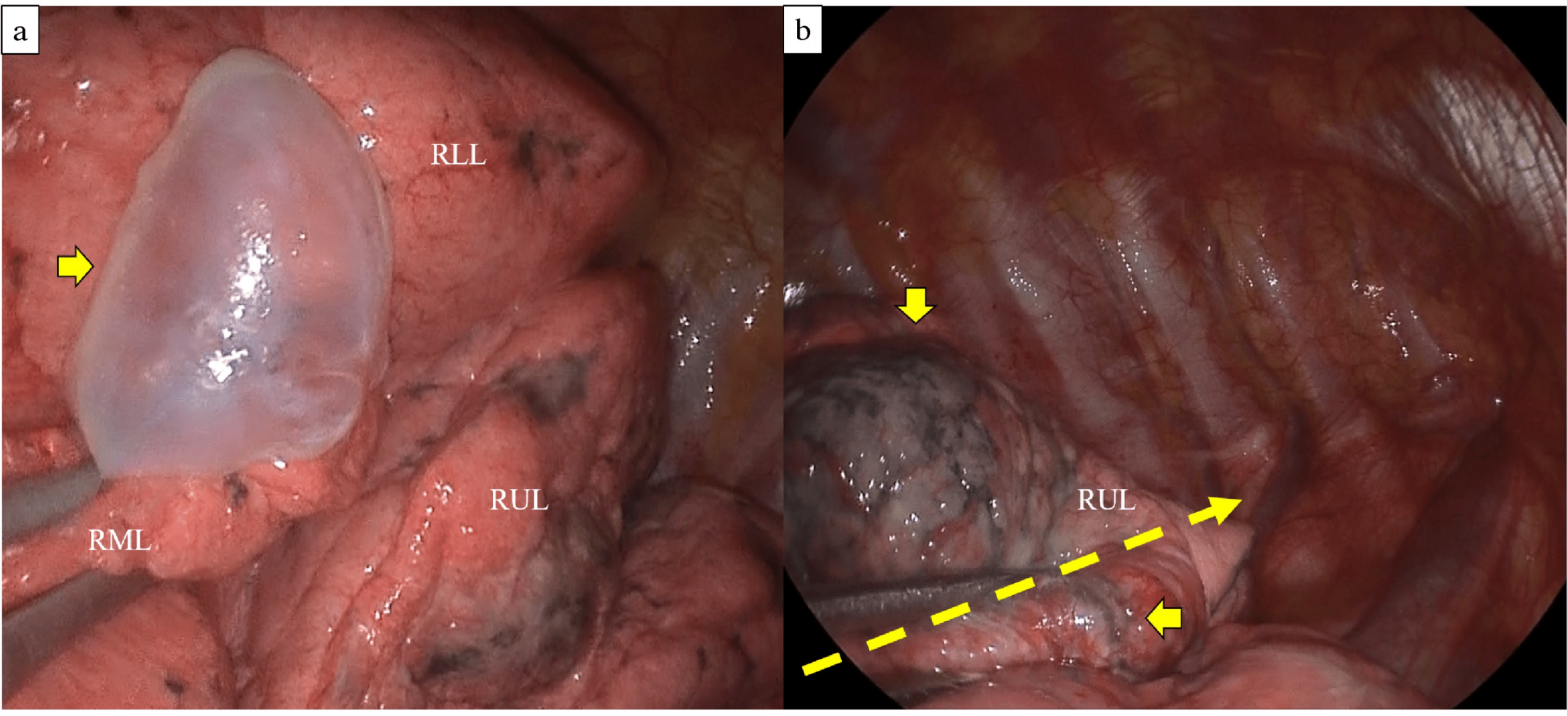
Surgical photographs a. A bullous lesion (yellow arrow) is visible in the right middle lobe; b. A grayish-white pleural change (yellow arrow) was observed in the right lung apex. Yellow dotted arrow depicts the lung resection line. RLL - right lower lobe; RML - right middle lobe; RUL - right upper lobe

**Figure 4:**
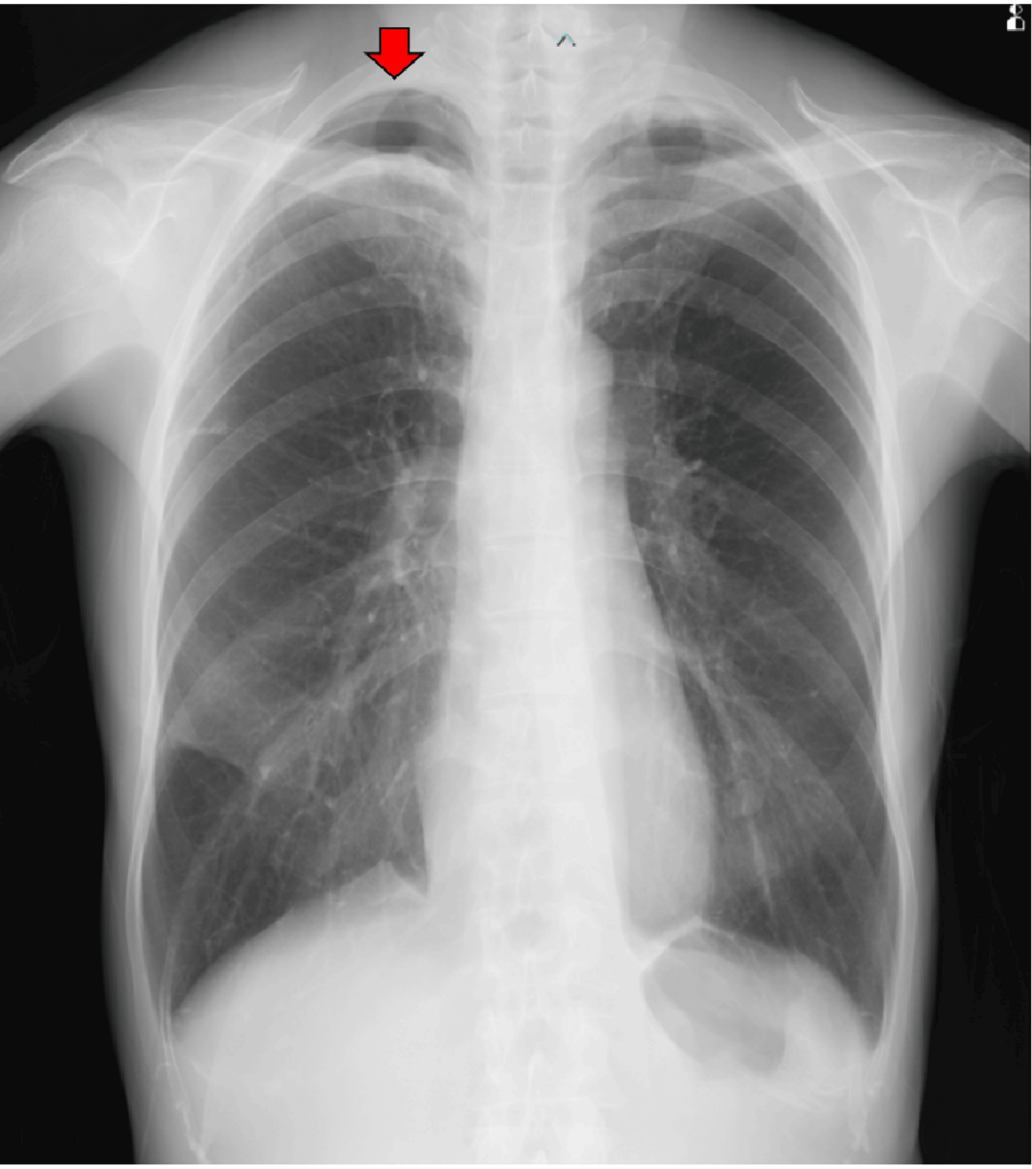
Chest radiograph on postoperative day five A space (red arrow) is present in the right lung apex, but lung expansion is preserved

**Figure 5:**
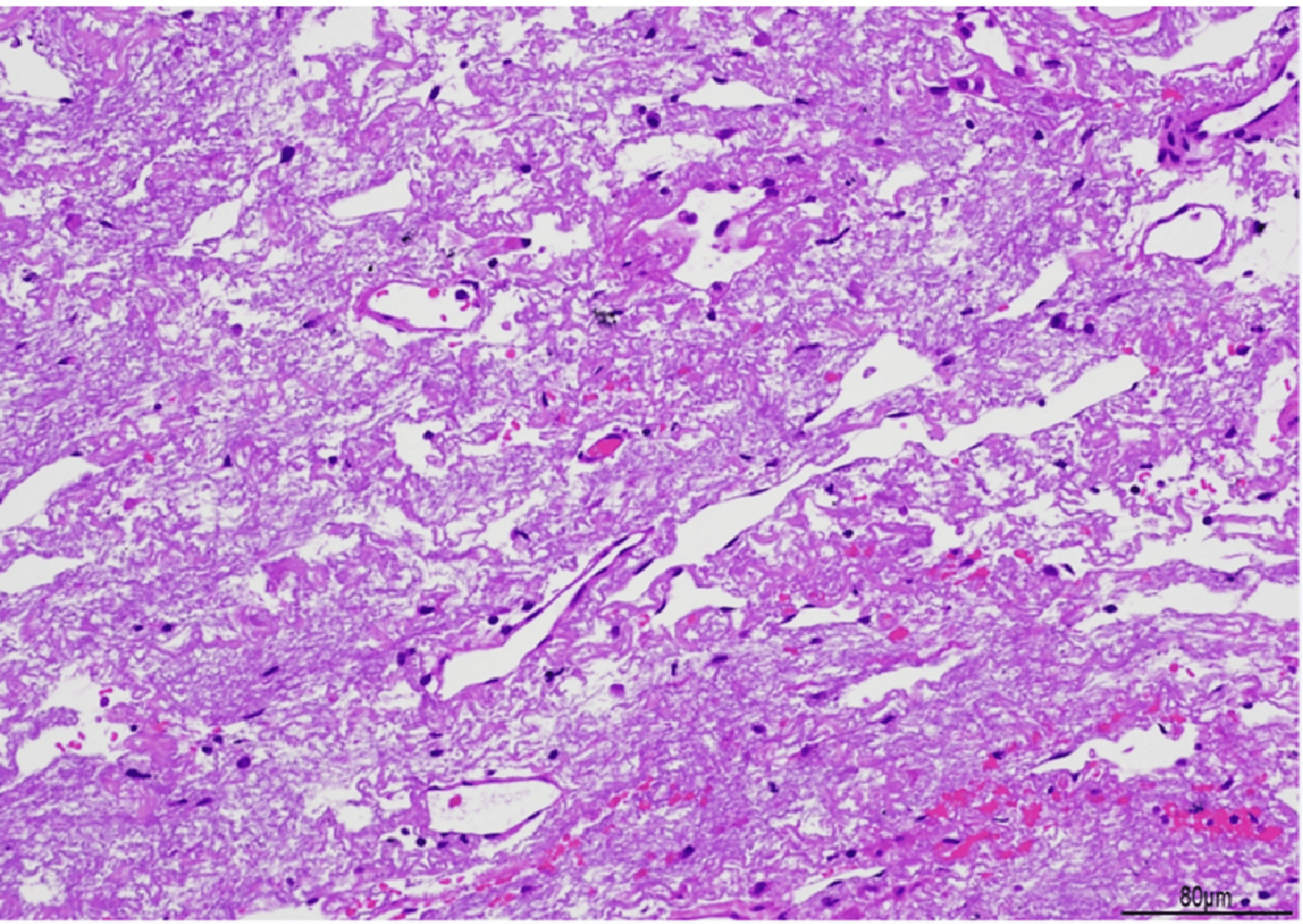
Pathological findings of pleuroparenchymal fibroelastosis Histology of hematoxylin and eosin-stained tissue from a partial resection of the right lung apex (magnification: 100x). Marked proliferation of elastic fibers and narrowing of the alveolar space were visible directly beneath the pleura. Collagen fibers are scarce, and no inflammatory cell infiltration is observed.

The patient was a 66-year-old non-smoking woman with no prior lung-related medical history who presented with a non-productive cough that had lasted for one week. Chest radiography revealed a severe right pneumothorax, and a chest drain (22 Fr) was inserted through the right fourth intercostal space at the anterior axillary line (Figure 1). CT revealed infiltrates in both the lung apices and a bulla between the right middle and lower lobes (Figure 2). Pulmonary function testing was not performed preoperatively because forced respiratory maneuvers might exacerbate the pneumothorax. Preoperative oxygen saturation was 97-98% (room air). No obvious air leakage was observed, but the patient requested surgical bullectomy; therefore, a single-port thoracoscopic right lung bullectomy was performed. A right middle-lobe bullectomy was performed after the bulla was identified intraoperatively. A grayish-white pleural change was observed at the right lung apex; therefore, a portion was partially resected for diagnostic purposes (Figure 3). No leakage points were observed. The blood loss was 2 mL, and the surgery duration was 44 min. On postoperative day (POD) one, the right chest drain was removed. The patient was discharged on POD six as no clear recurrence was observed on chest radiography (Figure 4). Pathological findings confirmed a right middle lobe bulla, and a biopsy of the resected lung from the right apex showed findings consistent with PPFE (Figure 5).

**Figure 6:**
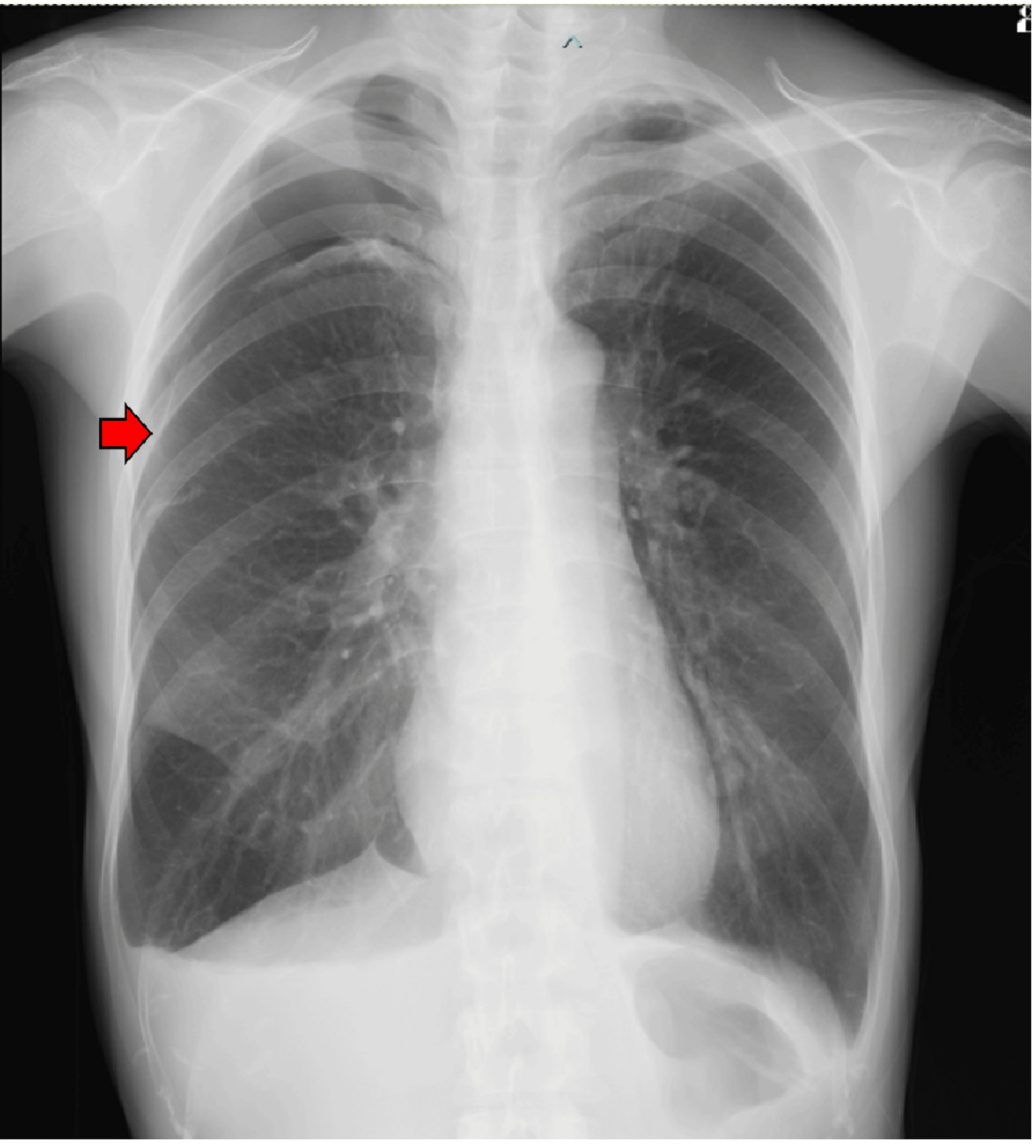
Chest radiography on postoperative day 16 Enlargement of the space in the right lung apex is visible, with progression of the right lung collapse (red arrow)

**Figure 7:**
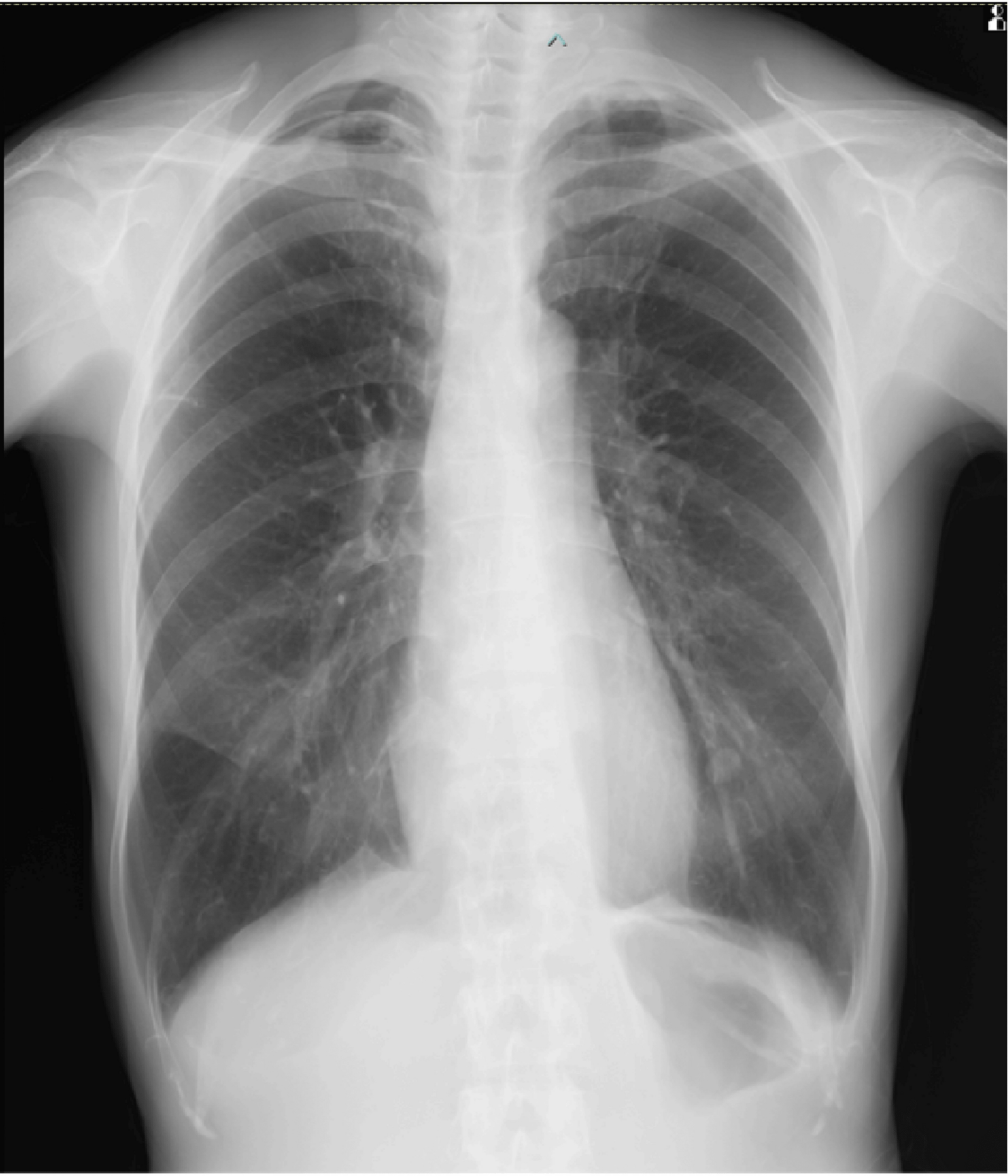
Chest radiography on postoperative day 30 Although space remained in the right lung apex, expansion of the right lung improved

On POD 16, a chest radiograph taken during the postoperative follow-up revealed progression of right lung collapse (Figure 6). This was diagnosed as a recurrence of right pneumothorax, and drainage of the right chest was repeated. Surgery was proposed because of recurrent pneumothorax; however, the patient refused. Owing to the patient's minocycline allergy, pleurodesis was performed on POD 20 using 50% dextrose solution (Otsuka Pharmaceutical Co., Ltd., Tokyo, Japan). Initially, the planned procedure involved intrapleural injection of 200 mL of 50% glucose solution in combination with 10 mL of 1% lidocaine. However, it was stopped after 150 mL was injected because the patient complained of shortness of breath. Two hours after the pleurodesis, the blood glucose level was 124 mg/dL. The post-pleurodesis pleural effusion volumes ranged from 315 to 1000 mL/day. The right chest drain was removed on POD 23, and the patient was discharged on POD 25. Chest radiography on POD 30 showed no clear recurrence of the right pneumothorax (Figure 7). The patient requested regular checkups for PPFE at a hospital near their workplace. The postoperative follow-up period at our hospital is 30 days. A timeline summarizing major clinical events, interventions, and outcomes is presented in Table 1.

**Table 1:** Timeline summarizing major clinical events, interventions, and outcomes POD - postoperative day

Number of days since the first consultation	Event
About seven days prior	Cough
1	First visit. Right chest tube insertion
9	Surgery performed
10 (POD 1)	Removal of the right chest tube drain
15 (POD 6)	Discharge
25 (POD 16)	Recurrence requiring right chest tube insertion
29 (POD 20)	Pleurodesis
32 (POD 23)	Removal of the right chest tube drain
34 (POD 25)	Discharge
39 (POD 30)	End of follow-up

## Discussion

During surgery, we suspected that the bulla was the cause of the pneumothorax and performed right middle lobe bullectomy. However, because recurrence occurred shortly after surgery, we diagnosed the patient with secondary pneumothorax recurrence and idiopathic PPFE. If an air leak had occurred preoperatively, an intraoperative water-sealing test might have been effective. However, no air leak was observed preoperatively in this case; therefore, we did not perform a water-sealing test before intraoperative resection. As preoperative CT revealed infiltrates in both lung apices, suggesting the possibility of PPFE, we performed a partial biopsy of the lesion in the upper right lobe apex. We resected only a portion of the lesion because a therapeutic effect could not be expected in this case, even if all lesions were resected, and a wide resection area would increase the postoperative dead space.

In the present case, the patient had no remarkable medical history and was diagnosed with idiopathic PPFE. Because this patient was allergic to minocycline, pleurodesis was performed using a 50% glucose solution. Pleurodesis with a 50% glucose solution is occasionally used for postoperative pulmonary fistulas or spontaneous pneumothorax [9,10]. It is repeated two to three times, with local anesthesia administered to the parietal pleura, followed by the injection of 200-500 mL of 50% glucose solution into the pleural cavity [10]. The success rate of pleurodesis with 50% glucose solution for air leaks after lung resection or pneumothorax has been reported to be 60.9% on the first attempt, 78.3% on the second attempt, and 84.8% on the third attempt [11]. In this case, the procedure was stopped after injecting 150 mL of a 50% glucose solution because the patient complained of shortness of breath. However, the pneumothorax improvement was observed after only one pleurodesis. The mechanism of pleurodesis using a 50% glucose solution is not yet fully understood. In a group of patients who successfully underwent pleurodesis using a 50% glucose solution, the amount of pleural effusion after lung resection significantly increased [11]. It has been hypothesized that differences in osmotic pressure damage pleural cells, leading to osmotic cytotoxicity, inflammation, and fibrin deposition, thereby completing pleurodesis [11]. The residual right apical space persisted despite lung expansion, likely because of partial resection and impaired expansion associated with apical infiltration. In this case, injecting a 50% glucose solution into the pleural cavity also temporarily increased pleural effusion owing to osmotic pressure differences, and the space in the right lung apex was replaced by pleural effusion. The replacement of the air space in the pleural cavity with pleural effusion may have contributed to the improvement of the pneumothorax.

In patients with pneumothorax, intraoperative pleural glucose spraying has been reported to cause elevated postoperative blood glucose levels and postoperative infections [12]. No adverse events were reported. In cases of PPFE where space remains in the lung apex even after right pleural drainage, as in this case, pleurodesis with a 50% glucose solution may be effective.

In addition to 50% glucose solution and minocycline, talc, autologous blood patch, and OK-432 are used in pleurodesis. However, talc administration for pleurodesis is restricted in Japan. For pneumothorax, talc is only covered by insurance for refractory pneumothorax. Intrapleural administration of OK-432 can cause fever [11], which would burden the patient. Complications of autologous blood patch are rare, but obstructive tension pneumothorax, pleurisy, and empyema (incidence 0-9%) due to thrombi in the chest drain have been reported [13]. Therefore, we chose a 50% glucose solution.

Here, we report a rare case of right recurrent pneumothorax complicated by PPFE that was treated with pleurodesis using a 50% glucose solution. However, there are limitations to definitively proving a causal relationship from a single case report. Further investigations are required to determine whether this procedure is successful in other patients.

## Conclusions

A blebectomy was performed for right pneumothorax, but pneumothorax recurred, leading to a diagnosis of secondary pneumothorax associated with PPFE. Pleurodesis with 50% glucose solution was effective for right-sided recurrent pneumothorax complicated by PPFE, in which a dead space remained in the right lung apex. It is possible that the temporary increase in pleural fluid volume, which replaced the dead space with pleural fluid, was effective in improving pneumothorax. Further investigations are required to confirm these findings.
